# Donation of peripheral blood stem cells to unrelated strangers: A thematic analysis

**DOI:** 10.1371/journal.pone.0186438

**Published:** 2017-10-25

**Authors:** Annelies Billen, J. Alejandro Madrigal, Katrina Scior, Bronwen E. Shaw, Andre Strydom

**Affiliations:** 1 Anthony Nolan, London, United Kingdom; 2 UCL Cancer Institute, London, United Kingdom; 3 UCL Research Department of Clinical, Educational and Health Psychology, London, United Kingdom; 4 CIBMTR (Center for International Blood and Marrow Transplant Research), Department of Medicine, Medical College of Wisconsin, Milwaukee, WI, United States of America; 5 UCL Mental Health Sciences Unit, London, United Kingdom; RAND Corporation, UNITED STATES

## Abstract

**Background:**

Donation of haematopoietic stem cells, either through bone marrow (BM) or peripheral blood stem cell (PBSC) collection, is a generally safe procedure for healthy donors, although side effects are a known risk. Previous research, including our recent quantitative study, has shown that the psychosocial response to donating is usually a positive one and most donors would be willing to donate again in the future. This is often despite experiencing significant side effects during the donation process. Due to the relative recent introduction of PBSC, a comprehensive understanding of the range of physical and emotional issues donors may experience is lacking, as well as an understanding of specific donor characteristics Qualitative research can provide rich narrative data into these areas. This study was set up in order to identify specific donor characteristics and to further explore the relationship between pre-donation physical health and the donation experience, as previously identified in our quantitative study.

**Methods:**

It involved in-depth telephone interviews with 14 PBSC donors who participated in our original quantitative study. Thematic analysis was used to analyse the findings and the results provide a summary of participants’ characteristics using themes and constituent codes.

**Results:**

We identified several donor characteristics, including strong intrinsic motivation, altruism, sense of duty, determination, low levels of ambivalence and the ability to develop a strong emotional relationship with an (unknown/anonymous) recipient whilst being able to manage strong feelings and emotions.

**Conclusions:**

These personality traits may explain the resilience that has been observed previously in haematopoietic stem cells donors. Significant feelings of grief were reported after a recipient’s death. Possibilities to alleviate these symptoms may include raising awareness of potential poor outcomes in the recipient and offering improved counselling services if the recipient dies. We acknowledge several limitations including the sampling frame.

## Introduction

Haematopoietic stem cell (HSC) transplantation is a curative procedure for life-threatening haematological diseases. During the past decade, peripheral blood stem cells (PBSCs) have replaced bone marrow (BM) as the main source of HSCs [1]. Ideally patients receive stem cells from a matched related (sibling) donor. But as there is only a 25% chance for a sibling to be human leukocyte antigen (HLA) matched, only about 30% of patients have a sibling matched donor [2]. An alternative donor source is an unrelated HLA matched donor that can be identified through volunteer unrelated donor registries. PBSCs are collected using a cell-separator machine connected to the donor via a peripheral cannula or a central venous catheter if donors have inadequate veins. Blood is taken from the donor and circulated in the machine where mononuclear cells are collected by centrifugation before the red cells are returned to the donor, a process called leukapheresis. This process usually takes 4 hours and a second day leukapheresis may be needed. Peripheral blood contains too few HSCs, hence growth factors (granulocyte colony stimulating factor or G-CSF) are injected daily the 4 or 5 days preceding the leukapheresis [3]. Although the donation process is generally safe, side effects are a known risk [4–9] and care must be taken to minimize harm to donors. Common physical side effects include bone pain, headache, nausea and fatigue. The psychological reactions following donation have been mainly studied in BM donors [10–12] and have been reported to be generally positive. Most subjects feel deep personal satisfaction and gratitude for an opportunity to donate [11].

We recently published a prospective quantitative study involving 275 PBSC donors examining health-related quality of life (HRQOL) during the donation process and examined factors that may be associated with recovery and side effects [13]. HRQOL was measured pre-donation, 4 weeks and 3 months post donation using the SF-36 questionnaire. The SF-36 is a generic indicator of HRQOL derived from the 245-item Medical Outcomes questionnaire. The Physical Component Summary (PCS) and Mental Component Summary (MCS) scores provide a broad physical and mental health perspective [14]. Norm based scoring was used to interpret the different dimensions’ and summary scores [14]. This scoring is created by adjusting the 0–100 score by the general population’s average and standard deviation (SD). As a consequence, the population mean and standard deviation of all scores are 50 and 10 respectively with higher scores reflecting more positive health states. To the best of our knowledge this was the first study to show that, even in PBSC donors, physical health is still significantly (although minimally) reduced 4 weeks following donation compared to pre-donation. Physical health had returned to pre-donation values at 3 months following donation. Mental health remained high throughout in PBSC donors. Similarly, a recent NMDP (National Marrow Donor Program, USA) study reported declined physical status immediately following donation and then scores higher than baseline levels at 6 and 12 months following donation, both for PCS and MCS scores [15]. Despite the considerable impact of donation on HRQOL peri-donation, most donors decide to remain active on a donation register [13, 16].

Due to the relative recent introduction of PBSC, a comprehensive understanding of the range of physical and emotional issues PBSC donors may experience is lacking, as well as an understanding of specific donor characteristics. These are needed in order to ensure donors’ concerns are addressed. Qualitative research can provide rich narrative data into these areas. Some qualitative research in this area exists, although mostly in BM or sibling donors [11, 12, 17–21].

This qualitative study aimed to identify specific donor characteristics and to further explore the relationship between pre-donation physical health and the donation experience as previously identified in our quantitative study. It involved in-depth telephone interviews with 14 PBSC donors who participated in our original quantitative study [13].

## Materials and methods

### Participants

Fourteen unrelated PBSC donors (eleven male and three female) whose donation was facilitated via the local registry, Anthony Nolan (AN), between February and November 2013 and who had previously participated in the prospective study [13] were selected. The donors were selected based on their pre-donation SF-36 Physical Component Summary (PCS) scores, which had been returned by 197/275 (72%) of donors in the prospective study (13); seven out of 53 donors with scores in the lowest quartile (≤ 56) and seven out of 53 donors with scores in the highest quartile (> 60) were randomly selected and approached by AB. The rationale for this was based on the finding that lower baseline physical health scores (measured using PCS) were shown to be significantly associated with longer recovery and very common side effects such as fatigue and pain in our quantitative study [13]. Mean pre-donation PCS scores in our quantitative study were well above the mean of the general population and even donors with pre-donation PCS scores in the lowest quartile had scores within one SD of the mean, reflecting the strict medical assessment of donors. We will refer to “low” and “high” scores, but this should be interpreted relative to the donor group, not relative to the general population.

Donors were not selected for age or gender. Recruitment was complete when saturation on key themes was reached. Saturation was defined as the point in data collection when new data produced little or no change to the thematic framework process and analysis [22]. All selected donors were provided with written information for the study and gave verbal informed consent for the telephone interview. All consents were audiotaped and full ethical approval for the consent and other procedures were obtained from the Institutional Review Board for the Anthony Nolan.

### Interview schedule and procedure

Interviews took place between 1 and 1.5 years following donation using a semi-structured interview schedule designed by the researchers. All participants were interviewed over the telephone and audiotaped for between 25 and 70 minutes by the principal investigator (AB). Telephone interviews were chosen as higher response rates were anticipated compared to face-to-face interviews and in order to include participants from a wide geographical range. Donors were invited to recall and reflect in detail about HRQOL pre and post donation and their motivation to donate. Open semi-structured format questions were used flexibly, being omitted, adapted or elaborated according to the demands of individual interviews. Whilst trying to avoid directive or closed questions or interpretations, an approach of responding to the participants’ answers was adopted. In this way, questions were used to promote a two-way dialogue.

### Data analysis

All audiotaped interviews were fully transcribed, transcripts were anonymised and analysed. Thematic analysis was performed in line with Braun and Clarke’s outline of the process involving 6 phases (familiarisation with data, generating of initial codes, searching for themes, reviewing themes, defining and naming themes and production of a report) [23]. Detailed reading and re-reading of transcripts led to the generation of initial codes. Codes were generated in a systematic fashion across the entire data set. Subsequently, codes were combined under overarching themes. Guidelines to ensure the quality of the analysis were used [23], which included choosing themes that were internally coherent and consistent and without overlapping too much. All transcripts were initially coded by AB. Credibility checks were carried out by two co-authors (AS and KS) for quality control purposes, who each read and coded two transcripts at different stages of the process.

## Results

The characteristics of the participants (n = 14) are shown in Table 1. Participants 1 to 7 were selected based on low pre-donation PCS scores (≤56), participants 8 to 14, based on high pre-donation PCS scores (>60). Pre-donation Mental Component Summary (MCS) scores were not taken into account when selecting donors, but are represented on the table.

**Table 1:** Donor and HRQOL characteristics of qualitative study participants.

ID	Age	Gender	Ethnicity	Marital status	Depen-dants (n)	Blood donor	Pre-donation PCS score	Pre-donation MCS score
p1	40s	M	White Northern European	Missing data	0	N	45.33	56.8
p2	30s	F	White Northern European	M	≥ 3	Y	51.5	55.97
p3	40s	M	Jewish	M	≥ 3	N	47.45	50.64
p4	40s	M	White Northern European	M	Missing data	N	50.1	58.05
p5	60s	F	White Northern European	M	0	Y	48.92	61.13
p6	40s	M	White Northern European	P	≥ 3	N	49.3	56.97
p7	30s	M	White Northern European	S	0	Y	44.84	55
p8	40s	F	White Northern European	M	1–2	N	60.45	58.45
p9	40s	M	White Northern European	Missing data	≥ 3	N	60.62	58.97
p10	30s	M	White Northern European	M	1–2	Y	63.04	49.89
p11	20s	M	White Northern European	S	0	Y	61.88	54.31
p12	30s	M	White Northern European	Missing data	Missing data	Y	61.79	53.61
p13	20s	M	Other (White)	P	0	N	61.52	54.33
p14	20s	M	White Northern European	Missing data	1–2	Y	63.71	48.38

Marital status includes M(arried), S(ingle) or having a P(artner)

### Relationship between pre-donation physical health and donation experience

Our results illustrated the close link between poorer pre-donation physical state and a worse donation experience as previously reported in our quantitative study. Table 2 displays the donors’ responses when asking about their pre-donation physical health. Donors with lower scores (p 1–7) were very healthy in general, but often had mild limitations explaining their lower PCS scores. The main reason for scoring slightly lower than the other donors was often due to pain symptoms, usually back pain, limiting daily life activities to a minor degree. Only one donor (p4) reported considerable limitations in daily life due to back pain. Donors with higher scores (p 8–14) were in extremely good shape and were often marathon runners or engaged in physical exercise on a daily basis.

**Table 2:** Pre-donation physical health and physical and psychological reactions to the donation process.

Participant ID	Pre-donation physical health	Reaction to donating
p1	Knees little bit stiff when standing up for long periods of time (required for his job)	“I got an awful lot of pain, it felt like a pressure in my tummy like maybe there was something going to burst”
		He took time off work (he was advised not to work because of symptoms)
p2	Mild symptoms back and joints interfering with some activities like bending	“I had quite a lot of headaches, they lasted for a couple of weeks”
		She had to cut down on social activities
p3	Back pain, interfering with high impact sports (running and squash)	Quite a lot of bone pains during injections and feeling low
		He worked from home around the time of injections and the day after donation
p4	Back pain, on tramadol and codeine pre-donation, interfering with daily activities	“I developed bad hip pain and needed a shot of morphine”
		He took the week off (this was decided beforehand)
p5	Chronic back pain, on morphine patches, but very active lifestyle (Swimming and gym 3 times a week)	“I developed quite a lot of pain in my bones”
		She had to take more rest and take everything slower as it was very painful
p6	In very good health, very active	Low in mood and quiet following donation for some time
		He took it easier around the time
p7	Very active, cycles to work every day	“I developed severe back pain and was advised to go to A&E for pain control”
		He was signed off from work during the donation process and took it easy
p8	Skiing, surfing and daily cycling	“The headaches were horrendous. I was cranky and less patient and I shouted more easily”
		Significantly impacting work and social life
p9	Mountain bikes a lot	Fatigue following donation
		He went mountain biking after work the day after donation
p10	Very fit, recently ran a marathon	No symptoms at all, he continued to play cricket all day during the injections
		“It was like a day out in London really”
p11	Very healthy and active with no limitations	Limited back pain, it didn’t restrict normal activities at all
p12	Fitter than most other people	Bit of sore back not impacting work or social life
p13	Healthy and fit	Bit of stiffness for 2 hours with no impact on work or life whatsoever
p14	Plays rugby 3 times a week	Bit of a cold, but not sure it was donation related
		“It was almost identical to platelet donation, instead of one needle, there are two”

Donors were asked about their worst symptoms, physical or psychological, during the donation process. Donors with lower scores experienced more severe symptoms in general. These symptoms often significantly influenced daily work and social life. Two donors mentioned low mood as their most serious side effect following donation (p 3 and p7); both had lower pre-donation PCS scores. Donors with higher scores generally had an excellent donation experience with limited impact on their daily life.

### Thematic analysis

The thematic analysis results provide a summary of participants’ characteristics under five themes and their constituent codes (Table 3). S1 Table displays the first draft of the results of the thematic analysis. Minimal changes were made following the credibility checks by AS and KS and following a meeting between AB, AS and KS to resolve minor differences in coding structure (Table 3). Similar themes were observed in donors with low (p1-p7) and high (p8-p14) pre-donation PCS scores and hence no distinction has been made in the presentation of the results. Codes are illustrated with interview extracts, text omitted from quotes is denoted by (…).

**Table 3:** Themes and codes.

Themes	Codes	Number of interviews where theme emerged
**Intrinsic motivation**	Altruism as a personality trait	13
	Opportunity to save someone’s life	8
	Promotion of donation	7
	Donation precipitated by personal circumstances	5
	Hoping to rely on others’ donations if ever in need	4
	Religious identification and the decision to donate	3
	Community sense and the decision to donate	2
**Determination**	Sense of duty	7
	Playing down side effects	5
	Not influenced by other people	4
	Worries about not being able to donate	3
**Relationship with recipient**	Wanting to know the outcome	14
	Remaining realistic about possible outcome for the recipient	8
	Emotional reactions to donation process	7
	Fantasising about recipient	3
	Reactions of grief when finding out recipient has died	1
**Strong feeling of fairness**		3
**Ease of decision**		4

#### Theme 1: Intrinsic motivation

Seven codes illustrate the intrinsic commitment donors shared.

Altruism as a personality trait

The central importance of altruism as a personality trait was evident and altruistic acts appeared to be fairly typical for most donors.

“I have been giving blood since I was 18. (…) And so, it was just quite a normal thing to join, that kind of made sense.” (p2)

“You have to help others, don’t you? If you see someone lying on the street, you wouldn’t walk away, would you? (…) You have to do it for others, without knowing who they are.” (p6)

Two donors commented on feeling guilty about not being blood donors prior to their experience of PBSC.

“I have been a blood donor several years ago. I found it very uncomfortable. I should have persisted really but I didn’t. I feel guilty about it.” (p1)

Several donors commented on how they did not expect anything in return, highlighting a true selfless motivation of joining the register.

“I was meant to write a card, but I don’t want to put an expectation onto the recipient or any kind of thanks.” (p2)

“I didn’t do it to get a congratulation or to get a well done. I just wanted to help somebody.” (p14)

Opportunity to save someone’s life

Participants viewed donation as a chance to save someone’s life and an opportunity to show kindness and help another person. Of note, several stated that they felt “honoured” to have been selected as donors.

“At the time of the letter, I felt very blessed, blessed to give someone the chance.” (p8)

Hoping to rely on others’ donations if ever in need

Some donor statements implied the relative costs to themselves and benefits (to themselves or the recipient) of donating and expressed hope that should they or their family were in a similar desperate situation others would do the same for them.

“I think it is a bit like giving blood. You give blood because you know one day you hope that if something happened to you someone will be there to help you out” (p7)

“I asked myself a couple of very simple questions: If this were one of my children, would I not want someone to do whatever they could to save my child? (…) In this world, you can’t get by without helping each other and if we are not able to take that step, why the hell would someone else in your situation do it?” (p3)

Religious identification and the decision to donate

While many donors reported altruism to be a motivating factor, some saw themselves as belonging to specific social groups that impacted their decision to donate. Religious beliefs were cited by three donors as a major influence in deciding to donate.

“And I think, as a Christian, you have to go through these kind of things. It says in the bible: treat people as you want to be treated and I would hope someone would do the same for me. (…) The only thing necessary for the evil to prevail is for good men to stand by and do nothing” (p7)

“I am a Christian. I teach girls in a catholic school and teach them to try and save lives” (p8)

Community sense and the decision to donate

Other donors described how being part of a cultural community inspired them to become a donor.

“I live in a closed community. I joined the register because someone in the local community needed a donor. I often read in the local newspapers to see if they are looking for new donors.”

Personal circumstances

Rather than general altruism being the main motivating force, some donors viewed their donation as precipitated by specific personal circumstances. These donors tended to relate to the recipient and have empathic feelings towards them.

“My son had meningitis and there was nothing I could do. Knowing what it is like to have a sick child, I thought, it must be terrible for a parent to be in a similar sort of situation and not being able to help.” (p14)

“I was thinking of my children and of how I almost lost my son. I really related to the woman (in need of a transplant), as she might be a mom, she might have children!” (p8)

Promotion of donation

The intrinsic motivation to donate was reflected in the finding that many donors remained actively involved in the local registry’s recruitment events and fundraising activities.

“I organised an AN recruitment event to about 3000 people in the local area following donation. I have sent e-mails to AN saying that I am happy to be an ambassador.” (p3)

“I am so passionate about people joining. I tell people that you can also donate via the PBSC method and they are surprised. If that was better communicated, far more people would join.” (p13)

#### Theme 2: Determination

Sense of duty

Some donors felt that donating was almost an obligation or duty. Not donating was simply not an option, regardless of the difficulties they might encounter as a donor.

“Not for a second did I think, do I fancy this? (…) I couldn’t live with myself if I didn’t go ahead. I just wouldn’t be able to live with myself.” (p1)

“I suffer from needle phobia. It has been impossible doing blood tests in the past. My wife had to take my hand during the donation process. They gave me one or maybe two valiums as well. For this cause, I was willing to take on the challenge of the needle phobia.” (p3)

Not influenced by other people

Even though most donors felt encouraged and supported by their family or friends in their decision to donate, several donors commented that they made the decision independently, without being influenced by other people. Some family members expressed worries and concerns, but this did not influence the donor’s decision to donate.

“My wife was worried about something going wrong, but I have the (donor) card in my wallet and that is my final decision.” (p6)

Worries about not being able to donate

When donors were asked about concerns related to the donation process, several responded they were mainly worried about not being able to donate, rather than feeling worried about their own health. They were worried about the possibility of becoming unable to donate due to health reasons, or not being a good match for the recipient.

“I was concerned that I needed to be in better physical health. I was wondering whether the medical would show anything that would stop me donating. That would have been devastating to me.” (p3)

“I was hoping I would still be able to continue with the injections (despite the tonsillitis), but it was fine in the end.” (p7)

Playing down side effects

Despite the fact that some donors experienced considerable side effects, they did not perceive them as having a large impact after completing the donation.

Despite experiencing severe headaches up to a week following donation:

“I think I was quite lucky with the side effects” (p3)

Despite experiencing considerable pain:

“All I had to do was sort of roll up my sleeve and have a needle in my arm for a couple of hours. That was all that was to it.” (p1)

Despite having been advised to go to A&E for pain control:

“It wasn’t bad at all, I just saw it as a bit of pain that was related to the process” (p7)

#### Theme 3: Development of relationship with recipient

Emotional reactions to donation process

Most donors reported experiencing intense emotions in response to the donation process. Both positive and negative experiences were reported.

“When I found out I was a match, I was excited, but at the same time upset and sad, as it meant there was someone going through this all. So very mixed emotions, but at least knowing that potentially I could help that person outweighed the more sad feelings at the end of the day” (p13)

“I felt low after the donation and went very quiet for several weeks afterwards.” (p6)

One donor reacted quite differently and commented that he experienced some closure after the experience.

“Whenever I donated stem cells, I finished in my mind, it was over. And then I had to give more a couple of months later and that was very difficult, because in my mind I had made some closure. I was happy to do it again, but when I finished I thought I am glad this is over.” (p11)

Wanting to know the outcome

Most interviewees wanted to know as much as possible about the person who received their cells. Donors showed a special personal interest in their recipients and were concerned about them. Although realising the potential of receiving bad news, all donors asked to be informed about the outcome of the donation for the recipient. One donor commented that knowing less about the recipient would help him cope better with a potential recipient death, whereas another donor commented that having more information relating to the circumstances of a recipient’s death might help him manage his grief.

“I think I wanted to know more whether it worked or not rather than who it was. Maybe it would upset me more if it was for a small child and if it hadn’t worked…” (p12)

“I don’t like the unknown, good or bad, I want to know.” (p14)

“It would be easier to deal with the recipient’s death if you knew more about the person. You would want to know whether someone is depending on them.” (p6)

Fantasising about recipient

Several donors fantasised about recipients’ characteristics and personality traits and some wondered if they shared traits with the recipient.

“I have related a lot to that woman. We were of the same weight and she was one year older than me. And a woman. And I just thought, she might be a mum! She might have children. (…) I thought I hope her children are fine.” (p8)

“I was told it was an adult male and a male can be from 16 up until whatever age. I got a thank you card and I was wondering: Is he a 16–18 year-old and is it his mum or someone aged 40 or 50 and his wife wrote the card?” (p6)

Reactions when recipient dies

Given this background, it is not astonishing that donors are affected by recipients' deaths. Only one donor in this cohort was told that his recipient had died. This donor expressed feelings of grief.

“It was pretty devastating. It is a strange feeling. Somebody passing who you don’t even know but somehow you have a major connection. That’s why maybe I have been quite a bit upset since I was notified several months ago.” (p3)

This donor also commented on the lack of preparedness and the lack of emotional support or counselling following the news.

“Going through a test is easy but finding out bad news is not. I am not sure I was sufficiently prepared. I was notified by letter and maybe it would have been better to have spoken to somebody. Some degree of counselling would have been good. I understand time and funds are limited, but part of the aftercare for the donors should maybe be looked into.” (p3)

He nevertheless explained that he perceived the attempt to save the recipient’s life as worth the effort and noted a wish to donate again in the future.

Remaining realistic about possible outcome for the recipient

Even though most donors experienced strong emotions, they often demonstrated a capacity to manage strong feelings and impulses and their thinking remained rational.

“Actually you have given them quite a chance, because if you hadn’t done anything their chances of survival would have been depleted even more. (p7)

“If I hadn’t been on the register, the next one wouldn’t have been as good (a match), so the chances of survival would have been even less. In my mind, they had the best chances” (p10)

“The news my donor didn’t survive was pretty devastating. But what I did prolonged his life and it was maybe for a bleeping moment, a ray of hope for his family.” (p3)

#### Theme 4: Strong feeling of fairness

Donors who did not always feel supported by work, felt frustrated or upset. They felt it was not right to be treated in this way given the altruistic gesture they had shown.

“Work was not generous and didn’t give me the time off. I was very upset with a catholic school dealing with it so poorly really. The whole value of caring, really…” (p8)

“My old employer was not supportive, that was probably part of the reason why I left them. (…)They offered me unpaid leave, I felt that was wrong.” (p13)

#### Theme 5: Ease of decision

No conscious motivation

Some donors seemed to have decided to donate “automatically” without careful thought about the potential costs of donation or even their own motivations in becoming a volunteer.

“Joining the register was as simple as buying a packet of crisps. You buy it and you don’t really look at the packet thinking did I really need to buy it, you know, you just open them and eat them. That’s it.” (p1)

## Discussion

This qualitative study acknowledges the link between pre-donation physical wellbeing (measured using the PCS score) and the donation experience, as previously identified in our quantitative study; donors with PCS scores in the lowest quartile often experienced more severe symptoms. However, the donation experience was very positive overall in both donors with pre-donation PCS scores in the lowest and highest quartile. Previous studies have highlighted that the vast majority of PBSC and bone marrow donors indicated they would be willing to donate again in the future [13, 24, 25]. Nevertheless, a delayed recovery has been described in bone marrow donors compared to PBSC donors [7, 9, 16, 25].

We used thematic analysis to explore participants’ characteristics. We found several donor traits that may explain the donor resilience previously observed in HSC donors. Indeed, most donors had a very positive recollection of the donation experience despite some experiencing significant side effects. Also, all donors in this cohort were willing to donate again in the future. Despite our previous finding that donors with lower pre-donation PCS scores experienced more side effects, similar themes were observed in donors with low and high pre-donation PCS scores. We found that most donors were driven by a strong, intrinsic motivation to donate. Character traits such as altruism appeared to be a major determinant in the decision to donate. While intrinsic motivation does not occur in isolation and is in part a response to a public or social role it appeared that many interviewees were motivated by a genuine desire to help others. It has been suggested that donation to strangers may be considered an example of more extreme altruism than that to family members [26]. Four donors implied the awareness of the relative costs to the donor themselves and benefits (to themselves or the recipient) of donating, what has been called an ‘exchange-related motive’ [19]. Others viewed donation as a chance to save someone’s life, help another person and an opportunity to show kindness and help another person. Valued social identities were closely implicated in the decision to become a donor. These identities included religious connections (based on the belief that helping is emphasised by religion) and community sense. Some donors were motivated by past personal events (such as the illness of a child) and these donors appeared to be motivated by feelings of empathy towards their recipient. Four donors commented on how they almost joined the register “automatically” without giving it much thought. Switzer et al described this kind of motivation as an “idealized helping” motivation [19]. The types of donor motivations in our study are comparable to the donation motives that have been previously reported in NMDP bone marrow donors [19]. This is not surprising, as donors on the Anthony Nolan register agree to donate stem cells by either method at the time of joining the register.

Donors appeared extremely determined in their decision to donate and donation even seemed to be a moral duty to some. The decision to donate was largely made without deliberation with family members and friends and several donors commented on how they were not influenced by others in making the decision. The majority of participants in this study were white Northern European and these findings contrast with an Asian study, where decisions were often influenced by peers and friends [17]. This dedication to donation was also illustrated by the finding that several donors were worried about not being able to donate, more so than expressing worries about potential side effects or risks to their own health. If side effects were experienced, they were perceived as being minimal. This may also be a consequence of the retrospective nature of this study, as donors were interviewed 1 to 1.5 years following donation.

The strong intrinsic motivation and determination observed reflects very low levels of ambivalence. Ambivalence involves feeling unsure about donation and has been extensively studied in bone marrow donors by Switzer et al [15, 26]. High ambivalence encompasses the feeling of relief if donors find out they cannot donate, doubts and worries about donating or wishing that the patient was getting the stem cells from someone else. Switzer et al. found that low levels of ambivalence was the best predictor of positive donation-related outcomes [26, 27]. The same author showed that respondents with lower intrinsic commitment to join the donor registry (those who did not feel morally obligated to join and those who would not have been disappointed in themselves had they not joined) reported higher ambivalence. Donors who had been encouraged to or discouraged by others from joining the registry reported higher ambivalence as well as donors who believed they were not well informed about the donation process. These results are consistent with the low levels of ambivalence and the generally positive donation experience observed in this study, both in donors with low and high pre-donation PCS scores.

Even though donors are not related to the recipients, a strong emotional bond was often observed. In accordance with World Marrow Donor Association guidance, unrelated donors are permitted to find out about the outcome of the donation and to exchange letters anonymously via the donor registries and transplant centres. Two years after donation, anonymity between donors and recipients can be removed if both sides agree and no legal restrictions of the recipient country require on-going anonymity [28]. Similar to previous studies [28, 29], all donors interviewed wanted to know the outcome for the recipient. Only one donor in this cohort received bad news and he experienced significant symptoms of grief as a consequence. These unexpected, intense feelings of grief have been described previously [28, 30]. The explanation for this strong affective response may be due to the strong emotional relationship most donors develop with their recipient, even if unknown to them. Another explanation may be that the donor’s altruistic act has failed, leaving them disappointed. One might argue that donor centres could avoid such negative consequences for donors by not informing donors about recipients' deaths. However, there is general agreement that donor centres should inform donors who request this information on the health status of their respective recipients. This also applies to information about recipients' deaths [31]. One study also reported that donors preferred the knowledge of the failure to uncertainty, even if this knowledge caused grief [30]. Donors' hopes regarding the positive outcomes of transplantation may be unrealistic. It is therefore important that registries or collection centres provide realistic information during donation preparation and inform prospective donors openly about the possibility of failure. One author reported that donors who felt poorly informed prior to donation were significantly less likely to consider the message regarding the recipient's death to be helpful and informative [28]. The way this information is communicated is important–of note, most donors prefer to be contacted by telephone [29]. Registries should be encouraged to implement this and the role of trained counsellors in this respect should also be considered. The relevance of personal coping resources and referring donors to their family and friends has also been suggested [28]. Some registries have reported that sharing experiences with other donors may be therapeutic and therefore opportunities for donors to meet within a support group may be beneficial. Despite the death of a recipient, most donors in previous studies were happy to have donated and said they would be willing to donate again [28]. Even though an emotional bond with the recipient is often developed, this study found that donors seemed able to manage strong feelings and emotions, an important prerequisite to resilience.

A limitation of this qualitative study lies within the donor recruitment method, as only donors with PCS scores in the highest and lowest quartile were selected. This may limit the ability of these results to be generalized to the rest of the donor population, but this approach was used to seek greater insight through comparison of these subgroups. Our recruitment method did not include gender in the sampling framework and only 1/7 participants in the highest and 2/7 participants in the lowest quartile sample were female. These numbers reflect the total group, in which 10/52 with PCS scores in the highest quartile and 16/52 with PCS scores in the lowest quartile were female. The small number of female participants limits the exploration of female versus male views and we would hence argue that data saturation was not reached within our female donor group. Another potential limitation is the epistemological position that was adopted; one of essentially accepting what participants say as a genuine reflection of their experience. For example, donors may not have been consciously aware of rewards gained from the donation process or perhaps not willing to acknowledge such rewards in an interview as thinking of themselves as highly altruistic and presenting themselves to the world as such.

Telephone interviews were used, as we anticipated higher response rates through this method and in order to include participants from a wide geographical range. Telephone interviews encompass several differences compared to face-to-face interviews. The physical presence of the interviewer in face-to-face interviews means that a range of non-verbal channels of communication are available and signs of misunderstanding or frustration may be more easily acknowledged. It may also be easier for interviewers to establish rapport. As a result the respondent might feel more comfortable reporting socially undesirable behaviours or attitudes. On the other hand, face-to-face respondents may report sensitive behaviours or attitudes less truthfully, since they will be more aware of the interviewer’s reaction to their answers than a telephone respondent would be. As a result, face-to-face respondents may be more likely to edit responses to appear in a more favourable light (referred to as social desirability bias) [32].

This qualitative study identified several donor characteristics, including strong intrinsic motivation, altruism, sense of duty, determination, low levels of ambivalence and the ability to develop a strong emotional relationship with an (unknown/anonymous) recipient whilst being able to manage strong feelings and emotions. These personality traits may explain the resilience that has been observed previously in HSC donors. It should be borne in mind that this study recruited donors who had already donated and thus can be expected to be different from potential donors presenting at recruitment or confirmatory typing (CT) stage. Switzer et al reported higher levels of ambivalence at earlier stages in the donation process [27, 33, 34] and identified this as the main risk factor for donor attrition or opting out of the register. The finding that most donors in this cohort joined as a result of an intrinsic commitment, rather than as a result of extrinsic pressures, means that strategies could be considered to create a recruitment context that emphasises the former. Some strategies have already been considered by the NMDP and include creating materials that reinforce the message of intrinsic and long-term commitment to donation and avoiding recruitment settings that may involve high levels of extrinsic pressure to join, e.g. recruitment drives in schools [27]. Strategies to enhance commitment at recruitment could also involve a two-stage process, where the decision to join the register needs to be reaffirmed. Other approaches may include making it easier for donors to opt out of the register. Although most of the donor characteristics in this cohort are related to inherent personality traits, several aspects of the donation process may help to build a “resilient donor population”. Possibilities include raising awareness of potential poor outcomes in the recipient and offering improved counselling services if the recipient dies.

## Supporting information

S1 Table Themes and codes—first draft. (PDF)
