# Supplementary material for: Donation of peripheral blood stem cells to unrelated strangers: A thematic analysis
Source: PLoS One. 2017 Oct 25;12(10):e0186438. doi: 10.1371/journal.pone.0186438 (PMC5656410; doi:10.1371/journal.pone.0186438)
Supplement: S1 Table — (PDF) [file pone.0186438.s001.pdf]

S1 Table. Themes and codes - first draft

Themes	Codes
**Intrinsic motivation**	Altruism as a personality trait
	Anthony Nolan (AN) ambassador
	Guilt about not being a blood donor
	Favour to be returned if they would end up to be in similar situation
	Connection between religious identification and the decision to donate
	Connection between community sense and the decision to donate
	Donation precipitated by specific personal circumstances
**Determination**	Not influenced by other people
	Perseverance
	Donors worried about not being able to donate, rather than expressing worries about own health
	Playing down side effects
**Relationship with recipient**	Very emotional reactions to donation process, both high and low
	Fantasising about recipient
	Reactions of grief when finding out recipient has died
	Wanting to know about outcome and recipient in general
	Despite strong (emotional) relationship with recipient, very rational behaviour
**Strong feeling of rightness/fairness**	
**Not expecting reward**	
